# Internet-based cognitive behavioural therapy for insomnia comorbid with chronic benign pain – A randomized controlled trial^☆^

**DOI:** 10.1016/j.invent.2024.100781

**Published:** 2024-10-14

**Authors:** K. Bothelius, S. Jernelöv, V. Kaldo, C. Lu, M.-M. Stråle, M. Jansson-Fröjmark

**Affiliations:** a Department of Surgical Sciences, Uppsala University, Uppsala, Sweden; b Centre for Psychiatry Research, Department of Clinical Neuroscience, Karolinska Institutet, & Stockholm Health Care Services, Region Stockholm, Stockholm, Sweden; c Division of Psychology, Department of Clinical Neuroscience, Karolinska Institutet, Stockholm, Sweden; d Department of Psychology, Faculty of Health and Life Sciences, Linnaeus University, Växjö, Sweden; e Department of Psychology, Uppsala University, Uppsala, Sweden

**Keywords:** Cognitive behavioural therapy for insomnia, Chronic pain, Internet-based interventions, Treatment engagement, Sleep disturbances, Mechanisms of improvement

## Abstract

**Background:**

Comorbid sleep disturbances are common among individuals with chronic pain, and Cognitive Behavioural Therapy for Insomnia (CBT-i) has proven effective for such individuals. Nonetheless, research on web-based CBT-i tailored for patients with both chronic pain and insomnia is limited. This study aimed to evaluate the feasibility and efficacy of internet-based CBT-i and to explore potential mechanisms underlying treatment outcomes.

**Methods:**

In this study, 85 participants suffering from comorbid insomnia and chronic pain were randomized into two groups: Internet-based CBT for Insomnia (ICBT-i) and Internet-based Applied Relaxation (IAR). Both interventions spanned eight weeks, supported by therapeutic guidance throughout.

**Results:**

Participation was modest, with an average module completion of 2.0 out of 8 for ICBT-i and 2.4 for IAR. Both interventions significantly alleviated insomnia symptoms on one of the insomnia measures post-treatment, without notable differences between them. Directly after treatment, IAR outperformed ICBT-i in reducing pain interference, anxiety, and in enhancing self-rated health, though these differences lessened at the 6-month follow-up. Potential therapeutic mechanisms may involve attenuating maladaptive sleep beliefs and augmenting sleep-related willingness.

**Conclusions:**

The study encountered low engagement rates, with approximately one-third of participants not completing any module. The limited efficacy of ICBT-i may be due to low treatment involvement, with few patients completing key techniques like sleep compression and stimulus control. Despite the low adherence, both interventions yielded post-treatment improvements in insomnia symptoms, but to establish internet-based treatments for insomnia as a viable option in chronic pain management, patient engagement must be improved.

## Highlights

• Internet-based interventions alleviate insomnia in chronic pain patients.
• Engagement challenges observed, with many participants not completing any modules.
• Treatment gains attributed to improved sleep beliefs and increased willingness.

## 1 Introduction

Chronic pain and sleep disturbances are both prevalent and debilitating conditions (Smith and Haythornthwaite, 2004), with chronic pain affecting approximately 20 % of the population (Breivik et al., 2006; Vos et al., 2016). Among individuals with chronic pain, over 70 % experience sleep disturbances (Sun et al., 2021). Those suffering from both pain and sleep disturbances report higher levels of pain, increased disability, and elevated levels of depression and anxiety compared to those without sleep disturbances (Husak and Bair, 2020).

Cognitive behavioural therapy for insomnia, CBT-i (Morin et al., 2006), has been demonstrated to be effective in treating comorbid insomnia (Geiger-Brown et al., 2015; Wu et al., 2015; Zhou et al., 2020; Hertenstein et al., 2022; Blom et al., 2024). Specifically, CBT-i has shown both short- and long-term benefits for sleep quality in patients with comorbid insomnia and chronic pain (Selvanathan et al., 2021; Climent-Sanz et al., 2022). Internet-based CBT-i, ICBT-i, has emerged as a viable alternative to traditional CBT-i (Zachariae et al., 2016; Simon et al., 2023). However, the efficacy in patients with concurrent chronic pain remains underexplored, as the application of ICBT-i in this population has not yet been extensively studied. Recent evidence suggests that ICBT-i significantly improves insomnia severity and various sleep parameters (e.g., sleep efficiency, sleep onset latency) more rapidly than applied relaxation therapy. Nevertheless, by the 6-month follow-up, both treatments demonstrated similar improvements in sleep, with no significant effects observed on pain-related outcomes (Wiklund et al., 2022).

Although CBT-i is widely regarded as the first-line treatment for chronic insomnia (Riemann et al., 2017; Qaseem et al., 2016), there is a need for effectiveness and implementation trials that examine potential barriers within routine care settings (Koffel et al., 2018). Such trials are crucial for supplementing the existing knowledge base, particularly regarding the diverse patient population typically encountered in standard care (Baglioni et al., 2020). Additionally, it has been challenging to demonstrate positive effects of computer-based interventions on patient-reported outcomes for individuals with chronic pain (Vugts et al., 2018).

Several theories suggest that cognitive processes sustain insomnia (Espie, 2002; Harvey, 2002; Lundh and Broman, 2000; Morin, 1993). The research on mechanisms of CBT for insomnia and its mediator effects is relatively sparse, but generally suggests that alterations in cognitive factors — such as modifying dysfunctional beliefs and attitudes about sleep — are more significant predictors of CBT-i effectiveness than factors such as hyperarousal (Altena et al., 2023). For instance, maladaptive beliefs have been shown to mediate symptom improvement post-CBT-i (Parsons et al., 2021), and a recent meta-analysis found reductions in maladaptive beliefs to be associated to improvements in insomnia severity (Nielson et al., 2023). Treating chronic pain with therapies aimed at acceptance has been shown effective (Lai et al., 2023). Acceptance is also linked to sleep quality in individuals with chronic pain (McCracken et al., 2011). In addition, in CBT-i, acceptance of sleep problems has been suggested as a mechanism of change (Blom et al., 2016a). In the context of insomnia co-occurring with chronic pain, research on treatment-specific mechanisms is very scarce, and further exploration of the underlying processes of therapeutic effects is advocated (McCrae et al., 2019). As outlined above, dysfunctional beliefs and acceptance are two plausible candidates.

Given the limited research on ICBT-i in the context of chronic pain, particular in real-world clinical setting, this study aimed to assess the feasibility and effectiveness of ICBT-i for patients with chronic pain and insomnia in a routine care environment at a university hospital pain clinic; to confirm that specific effects of CBT-i also are found in this setting and patient group, and to explore dysfunctional beliefs and sleep problem acceptance as potential mechanisms of treatment. It was hypothesized that ICBT-i would be feasible in this setting and lead to significant reductions in insomnia symptoms compared to an active control treatment seen as credible and relevant for patients with chronic pain.

## 2 Methods

### 2.1 Design, procedure and random allocation

In this RCT at a university hospital pain clinic, 85 participants with insomnia and chronic pain were randomized into two insomnia treatment groups. Evaluations occurred pretreatment, post-treatment, and at 6-month follow-up. The primary endpoint was at post-treatment. The study was ethically approved (Uppsala Ethics Review Board, Dnr: 2016/510) and registered (ClinicalTrials.gov ID: NCT03075683). Aiming for an effect size of 0.8 on the ISI with 80 % power and 0.05 significance level (Zachariae et al., 2016; Morin et al., 2006), the design required 30 participants per condition, accounting for 25 % attrition, thus totalling at least 80 participants. Randomization was conducted using a list pre-generated by the last author through random.org, secured and accessed by the first author. The study was non-blinded.

### 2.2 Participants

**Fig. 1:**
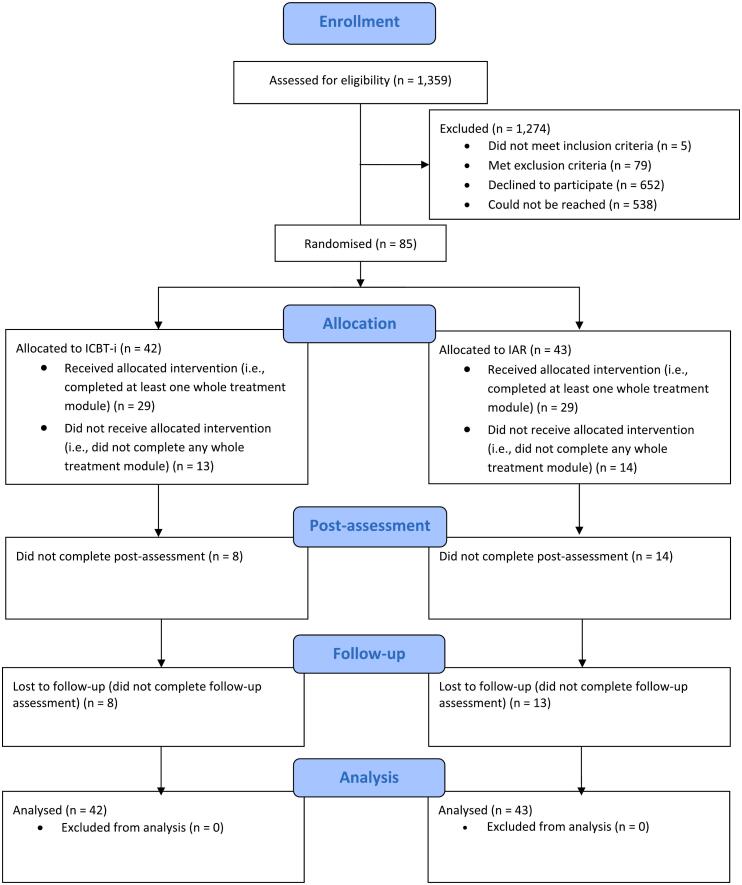
Flowchart of study recruitment.

Eligible patients were those who were visiting the pain clinic for the first time within a designated 90-week period. They were initially informed about the study through written communication, followed by a phone call for those who did not respond. Patients who expressed interest were provided with comprehensive study details, consented via a secure website, and completed a screening questionnaire. Subsequent assessments to determine eligibility were conducted through semi-structured telephone interviews. The inclusion criteria were as follows: adults aged 18 years or older, diagnosed with insomnia according to the American Academy of Sleep Medicine research criteria (Edinger et al., 2004), a score of over 10 on the Insomnia Severity Index (ISI) (Bastien et al., 2001), chronic benign pain (IASP, 1986), no pharmacological treatment for insomnia or pain, or stable treatment for at least two months, sufficient proficiency in Swedish, and the practical ability to participate in the study. Exclusion criteria included the presence of other comorbid disorders that required different treatments or could be adversely affected by sleep restriction, substance abuse, shift work or employment with high safety requirements for sustained attention, pregnancy, and prior receipt of CBT-i or current participation in a pain management program. The risk of substance abuse was assessed using the Alcohol Use Disorders Identification Test (AUDIT) (Saunders et al., 1993) and the Drug Use Disorders Identification Test (DUDIT) (Berman et al., 2005). Patients who were excluded from the study were referred to appropriate care as needed. After completing the pre-assessment, participants were randomly assigned to one of two conditions: ICBT-i or Internet-based Applied Relaxation (IAR), as well as to one of two therapists. See Fig. 1 for the flow-chart detailing the recruitment process of the study.

### 2.3 Measures

#### 2.3.1 Primary outcome

Feasibility was assessed through treatment adherence, gauged by the completion rate of modules per participant, alongside evaluations of treatment credibility and expectancy, which can positively influence adherence and feasibility. Treatment efficacy was evaluated using the Insomnia Severity Index (ISI), a 7-item, 0–4 graded self-report scale (total score range: 0–28, higher scores denoting greater insomnia severity), with adequate psychometric properties and sensitivity to change (Bastien et al., 2001).

#### 2.3.2 Secondary outcomes

Eight self-report scales were used as secondary outcomes to assess the effectiveness of ICBT-i: the Bergen Insomnia Scale (BIS), the Brief Pain Inventory-Short Form (BPI-SF), the Montgomery-Åsberg Depression Rating Scale – Self (MADRS-S), the Generalized Anxiety Disorder scale-7 (GAD-7), the Work and Social Adjustment Scale (WSAS), the Brunnsviken Brief Quality of Life Questionnaire (BBQ), the Clinical Outcomes in Routine Evaluation-Outcome Measure 10 (CORE-10), and the Self-rated health-5 (SRH-5).

The BIS is a validated tool for assessing insomnia, with scores ranging from 0 to 42 indicating severity; higher scores suggest greater impairment (Pallesen et al., 2008).

The Brief Pain Inventory-Short Form (BPI-SF), an 11-item questionnaire, assesses both pain severity (4 items, 0–40) and pain interference (7 items, 0–70), with higher scores indicating greater pain and interference). The BPI-SF is widely endorsed as a reliable outcome measure for chronic pain trials (Cleeland and Ryan, 1994; Dworkin et al., 2005).

The MADRS-S is a validated nine-item scale for depressive symptom severity (Svanborg and Asberg, 1994), showing high correlation with other depression scales (Svanborg and Asberg, 2001), and the expert-rated version (Mattila-Evenden et al., 1996). Scores range from 0 to 27, with higher scores denoting greater symptom severity.

The GAD-7 scale is a validated seven-item instrument that quantifies anxiety on a 0–21 scale, indicating severity (Spitzer et al., 2006).

Initially designed for generalized anxiety disorder screening (Lowe et al., 2008), the GAD-7 is now widely used in research and clinical practice due to a balanced assessment of cognitive and somatic symptoms and strong convergent and discriminant validity across various anxiety and mood disorders (Rutter and Brown, 2017; Johnson et al., 2019).

The WSAS, comprising five self-reported items, assesses functional impairment due to health issues, scoring between 0 and 40 where lower scores signify lesser impairment (Mundt et al., 2002). It demonstrates satisfactory psychometric validity in insomnia patients (Jansson-Frojmark, 2014).

Quality of life measurement employed the BBQ, a 12-item tool with scores 0–48, where higher scores denote greater life satisfaction, validated for psychometric reliability (Lindner et al., 2016).

The CORE-10, a ten-item scale, quantifies psychological distress with scores from 0 to 40, where higher scores indicate increased distress (Barkham et al., 2013).

Self-rated health-5 (SRH-5) employs a single-item scale for personal health assessment, ranging from 1 (‘very good’) to 5 (‘very poor’) (Eriksson et al., 2001). It possesses reliable psychometric qualities (DeSalvo et al., 2006), and effectively forecasts future health and mortality (Desalvo and Muntner, 2011).

#### 2.3.3 Putative mechanisms

Two self-report measures were employed to assess putative mechanisms: the Sleep Problem Acceptance Questionnaire (SPAQ) and the Dysfunctional Beliefs and Attitudes About Sleep Scale – 10 (DBAS-10).

The SPAQ, an eight-item tool with subscales for willingness (SPAQ WILL) and activity engagement (SPAQ ACT), scores from 0 to 48, where higher scores indicate greater acceptance of sleep issues. It demonstrates satisfactory psychometric validity (Bothelius et al., 2015).

The DBAS-10, a ten-item scale assessing insomnia cognitions, ranges from 0 to 100, with higher scores indicating stronger unhelpful beliefs. It is psychometrically sound (Espie et al., 2000).

#### 2.3.4 Treatment credibility, expectancy, and safety monitoring

Treatment credibility and expectancy was assessed using the five-item Credibility/Expectancy Questionnaire (CEQ) with 11-point items (0–10), totaling 0–50 points; scores above 30 suggest adequate credibility (Borkovec and Nau, 1972). The CEQ has been validated for psychometric quality (Devilly and Borkovec, 2000).

Data on adverse events (AEs) were collected using a standardized side-effects checklist and interference scale (Kyle et al., 2011). Participants were also encouraged to voluntarily report any harms or unintended effects during treatment sessions.

All outcomes were administered at pre-, post-, and follow-up assessments, except for the CEQ and AEs, which were assessed only at post-assessment.

### 2.4 Intervention

The ICBT-i program adheres to a rigorously researched manual (Jernelov et al., 2012; Blom et al., 2015b; Blom et al., 2015a; Kaldo et al., 2015a; Forsell et al., 2019; Kaldo et al., 2020) showing sustained effects after three and ten years (Blom et al., 2016b; Blom et al., 2017; Jernelov et al., 2022) which is also used in regular clinical practice at the Internet Psychiatry unit since 2017 (internetpsykiatri.se). The manual incorporates essential practices such as sleep restriction, stimulus control, and cognitive restructuring. The treatment for the ICBT-i group was organized into modules addressing various components of cognitive-behavioural therapy, including psychoeducation on sleep and insomnia, sleep diary tracking, sleep rhythm management, relaxation techniques, mindfulness, acceptance, thought management, and sleep hygiene. The intervention is tailored to chronic pain through clinical cases to exemplify how a patient with pain could anticipate and experience for instance going through sleep restriction therapy, and adjustments in the description of some techniques, for instance incorporating pain experiences in the description of relaxation techniques and acceptance exercises. None of the treatment techniques were fundamentally altered. ACT principles and exercises are incorporated in module four, focusing on accepting difficulties and non-perfection, and in module seven where more advanced techniques such as radical acceptance, are introduced. It parallels an internet-based applied muscular relaxation control, IAR, which emphasises the practical application of relaxation techniques (Da Cunha Meneses and Tingstam, 2015). The modules in this group covered extended relaxation, abdominal breathing, full-body relaxation, short and conditioned relaxation, and active and quick relaxation. Both interventions are structured into eight text-based modules delivered over eight weeks through a secure online platform, detailed in Supplementary Table S1. The first module is made available at the start of treatment, and each following module is made available after the patient has sent in the homework from the previous module. Treatment support for both groups was provided by six psychology students in their final year of training, all of whom had completed clinical training in CBT. These students received specialized training and clinical supervision from one of the authors (MJF), who also oversaw treatment integrity. To ensure consistency in treatment delivery, standardized protocols were used, and therapists received ongoing supervision and monitoring. Therapist activities were continuously recorded to maintain treatment fidelity throughout the study. Treatment support included feedback on home assignments, opportunities to ask questions, and motivational enhancement. Responses to messages were typically provided within one to two working days, and no additional treatment information beyond what was outlined in the treatment manual was given. Communication was primarily maintained through an integrated messaging system. To encourage adherence, participants who were inactive for seven consecutive days received a reminder via text message. If inactivity persisted for another day, a phone call was made, and if necessary, further attempts to contact the participant were made every second day for up to one month. Adverse events or unintended effects were documented if reported by participants during their interactions with therapists.

### 2.5 Statistical analyses

Statistical analyses were conducted using SPSS version 27 (SPSS Inc., Chicago, IL), employing an intention-to-treat methodology. Sensitivity analyses using all study variables at baseline (socio-demographic and clinical variables) were executed for each outcome measure and missingness (yes/no) before the main analyses were performed; none of the variables were significantly associated with the outcomes or missingness. The main analyses, as described below, were thus performed considering data missingness.

Treatment effects were assessed using multilevel modelling, initially to explore the overall effect of time (combining both groups), followed by the inclusion of a group variable to evaluate the group-time influences on continuous outcomes across treatment phases. In keeping with the intention-to-treat principle, all missing values at post-treatment and follow-up were imputed with multiple imputation using the pretreatment assessment. The two interventions (ICBT-I and IAR) were compared using two piecewise models – from pre- to post-treatment and from post-treatment to follow-up – to capture change during the active treatment period and during the period following the completion of the interventions. To correct for using two sets of models, the *p*-value was corrected by dividing the traditional p-value of 0.05 by 2 (Schulz and Grimes, 2005; Streiner, 2015); the p-value that was used as the criterion for statistical significance for the outcomes in this study was thus 0.025. Parameter estimation was achieved via the maximum likelihood method with an unstructured covariance approach. Analysis proceeded sequentially from a simple fixed intercept model, through models incorporating random effects (intercept and slope), to those including a group-time interaction, using likelihood ratio tests for model comparison.

The putative mechanisms were analysed in an identical manner as the primary and secondary outcomes, i.e., using multilevel modelling to explore between- and within-group changes from pre- to post-treatment and from post-treatment to follow-up. Traditional mediation analysis was not performed since the study did not pursue testing mediational hypothesis from the outset. Also, our small sample size would likely be underpowered to test indirect effects. Despite this, it is possible that the results from this trial may inform future study designs to conduct mediation analysis.

Cohen's d was employed to calculate within-group effect sizes from pre- to post-treatment and from post-treatment to follow-up (pretreatment minus post-treatment or follow-up/pooled standard deviation) as well as between-group effect sizes at post-treatment and follow-up (post-treatment or follow-up for ICBT-i minus post-treatment or follow-up for IAR/pooled standard deviation). Effect sizes were interpreted in line with guidelines: effect sizes of 0.2–0.49 were considered small, 0.5–0.79 as moderate, and >0.8 as large (Cohen, 1988).

## 3 Results

Out of 1359 eligible patients initially contacted, 85 participants were ultimately included and randomized into the two study conditions: 42 in the ICBT-i group and 43 in the IAR group. In Table 1, socio-demographic and clinical parameters are presented for the two groups separately. The average age was 48 years, with a higher percentage of females in the ICBT-i group (81 %) compared to the IAR group (61 %). Most participants were married, cohabiting, or had a partner, with higher education levels being fairly evenly distributed across both groups. Employment status varied, with a significant portion on sick leave or disability pension.

**Table 1:** Descriptive statistics for the 85 study participants.

		ICBT-i (*n* = 42)	IAR (*n* = 43)
Age, years [M (SD)]		48.8 (10.8)	47.7 (13.6)
Gender (% female)		81 %	61 %
Civil status	Married, cohabitant or partner	69 %	81 %
	Divorced or widowed	5 %	7 %
	Single	26 %	12 %
Education	Compulsory school	12 %	7 %
	High school	41 %	49 %
	College or university	38 %	42 %
	Other	10 %	2 %
Occupational status	Working or student	48 %	37 %
	Sick leave or disability pension	50 %	49 %
	Unemployed	2 %	7 %
	Retired	14 %	23 %
	Other	2 %	2 %

*Note*. ICBT-i = Internet-based Cognitive Behavioural Therapy for Insomnia, IAR = Internet-based Applied Relaxation. The percentages for occupational status do not add up to 100 %, due to several participants reporting multiple occupational roles.

### 3.1 Primary outcome measure

#### 3.1.1 Treatment adherence

Treatment adherence, measured as the average number of modules completed per participant, was low in both the ICBT-i and IAR cohorts, with participants completing an average of 2.0 out of 8 modules (SD = 2.5) in the ICBT-i group and 2.4 modules (SD = 2.9) in the IAR group. Statistical analysis indicated no significant difference in adherence between the two groups (*t* = 0.67, *p* = 0.508). Details on module completion are presented in Supplementary Table S2. Completion of all eight modules was rare, with only 4.8 % of participants (*n* = 2) in the ICBT-i group and 9.3 % (*n* = 4) in the IAR group achieving this.

Additionally, a substantial proportion of participants did not initiate any modules, with 31.0 % (*n* = 13) in the ICBT-i group and 32.6 % (*n* = 14) in the IAR group, showing no significant difference between the groups (χ^2^ = 0.29, *p* = 0.591). Non-initiation, regardless of treatment group, was significantly associated with lower baseline quality of life (BBQ) and activity engagement (SPAQ ACT). Specifically, non-initiators had lower quality of life scores [M = 30.2 (SD = 17.7)] and lower activity engagement scores [M = 8.6 (SD = 4.4)] compared to those who completed at least one module [quality of life: M = 46.5 (SD = 20.7); activity engagement: M = 11.3 (SD = 6.3)]. Other socio-demographic and outcome variables were not significantly associated with initiation status.

#### 3.1.2 Treatment effect

Descriptive statistics, including means, standard errors, and standard deviations, alongside effect sizes for both groups at pretreatment, post-treatment, and 6-month follow-up, are delineated in Table 2, Table 3.

**Table 2:** Estimated marginal means, standard errors, and effect sizes for the treatment outcomes and putative mechanisms – from pre-treatment to post-treatment.

	Group	Pre-treatment	Post-treatment	Time (both groups combined)			Time × Group			Within-group effect sizes: from pretreatment to post-treatment	Between-group effect sizes (95 % CI): post-treatment
		M (SD)	M (SD)	B	SE	p	B	SE	p	d	d
ISI	ICBT-i	19.1 (2.7)	14.1 (4.0)	−0.44	0.21	0.044	−0.06	0.14	0.645	1.49	0.14 (−0.29 to −0.56)
	IAR	19.2 (2.3)	13.6 (3.3)							2.00	
BIS	ICBT-i	28.6 (8.0)	20.8 (9.3)	−0.81	0.34	0.019	0.04	0.22	0.870	0.90	0.14 (−0.29 to −0.57)
	IAR	27.0 (9.1)	19.6 (8.9)							0.82	
Pain severity: BPI	ICBT-i	24.5 (4.9)	22.5 (5.2)	0.05	0.26	0.858	−0.25	0.17	0.155	0.40	0.61 (0.17 to −1.04)
	IAR	23.6 (5.6)	19.1 (5.9)							0.78	
Pain interference: BPI	ICBT-i	43.3 (14.9)	42.2 (15.1)	0.86	0.48	0.079	−0.97	0.31	0.003	0.07	0.38 (−0.06 to −0.80)
	IAR	47.0 (17.0)	36.3 (16.3)							0.64	
MADRS-S	ICBT-i	19.6 (7.6)	16.6 (7.8)	−0.17	0.28	0.555	−0.13	0.18	0.461	0.39	0.41 (−0.03 to −0.83)
	IAR	17.9 (8.2)	13.5 (7.5)							0.56	
GAD-7	ICBT-i	7.7 (5.1)	7.2 (4.0)	0.20	0.15	0.177	−0.24	0.09	0.010	0.11	0.89 (0.44 to −1.33)
	IAR	6.8 (4.9)	3.9 (3.4)							0.70	
WSAS	ICBT-i	23.8 (6.8)	19.9 (7.6)	−0.31	0.36	0.396	−0.08	0.23	0.730	0.54	0.03 (−0.40 to −0.45)
	IAR	24.4 (6.5)	19.7 (7.2)							0.69	
BBQ	ICBT-i	38.6 (21.4)	44.5 (19.4)	0.75	0.91	0.416	−0.15	0.58	0.794	0.29	−0.21 (−0.63 to −0.22)
	IAR	44.0 (20.0)	48.4 (17.7)							0.23	
CORE-10	ICBT-i	16.5 (4.9)	14.7 (5.0)	−0.02	0.26	0.937	−0.17	0.17	0.332	0.36	0.81 (0.36 to −1.24)
	IAR	14.3 (4.8)	10.7 (4.9)							0.74	
Self-rated health	ICBT-i	1.5 (0.5)	1.6 (0.6)	−0.05	0.04	0.249	0.06	0.03	0.048	0.18	−0.92 (−1.36 to −0.46)
	IAR	1.5 (0.6)	2.2 (0.7)							1.08	
SPAC ACT	ICBT-i	10.2 (4.7)	11.7 (3.9)	0.09	0.22	0.692	0.06	0.14	0.670	0.35	−0.31 (−0.74 to −0.12)
	IAR	10.8 (4.9)	12.9 (3.8)							0.48	
SPAC WILL	ICBT-i	9.5 (2.2)	9.7 (2.6)	−0.13	0.26	0.613	0.15	0.17	0.366	0.08	−0.52 (−0.95 to −0.09)
	IAR	9.5 (2.6)	11.2 (3.1)							0.60	
DBAS	ICBT-i	53.6 (16.4)	36.4 (10.7)	−2.03	0.85	0.021	0.30	0.54	0.578	1.27	−0.02 (−0.44 to −0.41)
	IAR	50.7 (18.8)	36.6 (11.6)							0.93	

*Note*. BBQ = Brunnsviken Brief Quality of Life Questionnaire, BIS = Bergen Insomnia Scale, BPI = Brief Pain Inventory-Short Form, CI = confidence interval, CORE-10 = Clinical Outcomes in Routine Evaluation-Outcome Measure 10, DBAS = Dysfunctional Beliefs and Attitudes About Sleep Scale - 10, GAD-7 = Generalized Anxiety Disorder scale-7, IAR = internet-based applied relaxation, ICBT-i = internet-based cognitive behavioural therapy for insomnia, ISI = Insomnia Severity Index, M = mean, MADRS-S = Montgomery-Åsberg Depression Rating Scale – self-rated version, SE = standard error, SPAC ACT = Sleep Problem Acceptance Questionnaire - activity engagement, SPAC WILL = Sleep Problem Acceptance Questionnaire - willingness, WSAS = Work and Social Adjustment Scale.

**Table 3:** Estimated marginal means, standard errors, and effect sizes for the treatment outcomes and putative mechanisms – from post-treatment to follow-up.

	Group	Follow-up	Time (both groups combined)			Time × Group			Within-group effect sizes: from post-treatment to follow-up	Between-group effect sizes (95 % CI): follow-up
		M (SD)	B	SE	p	B	SE	P	d	D
ISI	ICBT-i	14.1 (5.2)	−0.04	0.20	0.853	0.06	0.13	0.661	0.00	−0.22 (−0.65 to −0.21)
	IAR	15.4 (6.4)							−0.37	
BIS	ICBT-i	20.4 (8.8)	−0.07	0.24	0.763	0.10	0.15	0.489	0.04	−0.30 (−0.73 to −0.13)
	IAR	23.3 (10.2)							−0.39	
Pain severity: BPI	ICBT-i	23.4 (6.5)	0.09	0.13	0.494	−0.02	0.08	0.800	−0.15	0.39 (−0.04 to −0.82)
	IAR	19.8 (11.2)							−0.08	
Pain interference: BPI	ICBT-i	40.1 (14.9)	−0.82	0.40	0.046	0.56	0.25	0.033	0.14	0.07 (−0.36 to −0.49)
	IAR	38.6 (26.2)							−0.11	
MADRS-S	ICBT-i	16.7 (7.9)	−0.11	0.22	0.809	0.15	0.14	0.703	−0.01	0.09 (−0.34 to −0.51)
	IAR	16.0 (7.7)							−0.33	
GAD-7	ICBT-i	8.1 (5.3)	−0.07	0.14	0.599	0.14	0.09	0.111	−0.19	0.24 (−0.19 to −0.66)
	IAR	6.7 (6.4)							−0.57	
WSAS	ICBT-i	15.6 (8.8)	−0.47	0.30	0.119	0.26	0.19	0.178	0.52	−0.45 (−0.87 to −0.01)
	IAR	20.3 (12.0)							−0.06	
BBQ	ICBT-i	41.5 (18.6)	−0.74	0.40	0.072	0.36	0.26	0.178	0.16	−0.42 (−0.84 to −0.02)
	IAR	49.5 (19.6)							−0.06	
CORE-10	ICBT-i	14.8 (6.8)	−0.26	0.15	0.091	0.25	0.10	0.012	−0.02	0.14 (−0.28 to −0.57)
	IAR	13.8 (7.1)							−0.52	
Self-rated health	ICBT-i	1.3 (0.9)	−0.02	0.03	0.577	0.01	0.02	0.806	0.40	−0.60 (−1.03 to −-0.16)
	IAR	1.9 (1.1)							0.33	
SPAC ACT	ICBT-i	11.8 (6.4)	0.02	0.18	0.918	<0.01	0.12	>0.999	0.02	−0.33 (−0.76 to −0.10)
	IAR	14.0 (6.8)							0.21	
SPAC WILL	ICBT-i	14.1 (5.4)	0.46	0.16	0.006	−0.17	0.10	0.114	1.10	0.17 (−0.26 to −0.59)
	IAR	13.1 (6.3)							0.40	
DBAS	ICBT-i	33.9 (10.4)	−0.62	0.67	0.362	0.48	0.42	0.270	0.24	−0.74 (−1.18 to −0.30)
	IAR	43.0 (13.8)							−0.50	

*Note*. BBQ = Brunnsviken Brief Quality of Life Questionnaire, BIS = Bergen Insomnia Scale, BPI = Brief Pain Inventory-Short Form, CI = confidence interval, CORE-10 = Clinical Outcomes in Routine Evaluation-Outcome Measure 10, DBAS = Dysfunctional Beliefs and Attitudes About Sleep Scale - 10, GAD-7 = Generalized Anxiety Disorder scale-7, IAR = internet-based applied relaxation, ICBT-i = internet-based cognitive behavioural therapy for insomnia, ISI = Insomnia Severity Index, M = mean, MADRS-S = Montgomery-Åsberg Depression Rating Scale – self-rated version, SE = standard error, SPAC ACT = Sleep Problem Acceptance Questionnaire - activity engagement, SPAC WILL = Sleep Problem Acceptance Questionnaire - willingness, WSAS = Work and Social Adjustment Scale.

Table 2 reveals a time effect on subjective sleep problems (*p* = 0.44, not meeting the corrected significance threshold) [ISI (ICBT-i: d = 1.49; IAR: d = 2.00)] from pre- to post-treatment for both groups combined, along with a non-significant interaction effect (d = 0.14). From post-treatment to follow-up, both time and interaction effects for subjective sleep problems were not statistically significant, exhibiting negligible to small effect sizes as shown in Table 3.

### 3.2 Secondary outcome measures

Between pre- and post-treatment, a statistically significant time effect on insomnia-specific sleep issues measured by the BIS (*p* = 0.019) was observed without a significant interaction effect (see Table 2). Overall, two statistically significant interaction effects emerged: pain-related interference (BPI interference subscale, *p* = 0.003), and anxiety severity (GAD-7, *p* = 0.010), with IAR demonstrating statistically significantly more substantial reductions in pain-related interference and anxiety severity than ICBT-i. The effect sizes for these between-group differences ranged from small to large (d = 0.38–0.92). No statistically significant time or interaction effects were found for the other secondary outcomes.

From post-treatment to follow-up, analyses revealed a non-significant within-group time effect on pain interference (*p* = 0.046) for both groups combined, along with two statistically significant interaction effects (detailed in Table 3). Specifically, the ICBT-i group exhibited a statistically significantly better outcomes in pain interference (*p* = 0.033) and psychological distress (*p* = 0.012), attributed to stability in the ICBT-i group versus worsening in the IAR group from post-treatment to follow-up. The effect size for between-group differences in psychological distress was negligible. No statistically significant time or interaction effects were noted for the other secondary outcomes.

Concerning treatment credibility and expectancy, both groups achieved adequate ratings (ICBT-i: M = 31.9, SD = 10.6; IAR: M = 32.4, SD = 8.2), and there was no statistically significant difference between the two groups post-treatment (*t* = 0.17, *p* = 0.869). In the ICBT-i group, 57.9 % (11 of 19) of participants reported at least one adverse event (AE), compared to 37.5 % (6 of 16) in the IAR group, with no statistically significant difference between the groups (χ^2^ = 1.45, *p* = 0.23). The mean number of AEs was 5.53 (SD = 7.10) in the ICBT-i group and 4.31 (SD = 9.8) in the IAR group, with no significant difference observed between the groups (Student's *t*: *p* = 0.67; Mann-Whitney U: *p* = 0.20). The lack of significant differences may be attributed to the small sample size and high attrition rates.

#### 3.2.1 Completer analyses

Individuals were classified as treatment completers upon completing four modules or more and thus engaging with the principal interventions, namely sleep restriction and stimulus control, in the ICBT-i framework. Collectively, analyses comparing completers to the entire sample cohort indicate a trend where individuals who completed ICBT-i exhibited more favourable outcomes at follow-up assessment compared to those who completed IAR, although this difference was not observed at post-treatment, see Supplementary Table S3.

### 3.3 Putative mechanisms

Between pre- and post-treatment, a statistically significant time effect was found solely for insomnia-related beliefs (DBAS, *p* = 0.021), as shown in Table 2. No other significant time or interaction effects were observed across the three proposed mechanisms: willingness (SPAQ WILL), activity engagement (SPAQ ACT), and insomnia-related beliefs (DBAS-10). From post-treatment to follow-up, a statistically significant time effect emerged for willingness (*p* = 0.006) alone (*d* = 0.40; *d* = 1.10), with no other significant time or interaction effects for the three mechanisms.

## 4 Discussion and conclusions

This study evaluated the feasibility and effectiveness of ICBT-i in patients with chronic pain and insomnia at a university hospital pain clinic. Although the treatment showed satisfactory levels of credibility and expectancy, as reflected by SEQ scores above 30, treatment adherence was low, with only a small minority of participants completing all eight treatment modules. Approximately one-third of participants did not initiate any modules, and overall module completion rates in the ICBT-i group were lower compared to previous studies involving other patient populations (Blom et al., 2015b; Blom et al., 2015a; Kaldo et al., 2015a), This finding raises concerns about the feasibility of ICBT-i for this specific patient group. Additionally, non-initiation was significantly linked to lower baseline quality of life and activity engagement, while other socio-demographic and outcome variables showed no significant association with initiation status.

The study showed a reduction in insomnia severity from pre- to post-treatment for the total sample, and particularly in the secondary insomnia measure (BIS). However, this reduction did not reach statistical significance for the primary outcome measure after adjusting the significance level, and no specific effect of ICBT-i on insomnia severity could be confirmed. The lack of specific effects could be due to problems with feasibility and treatment engagement, discussed more below. Notably, IAR outperformed ICBT-i in improving pain interference, anxiety, and self-rated health. However, the disparity in pain interference between groups narrowed at follow-up, attributed to ICBT-i gains and IAR regressions, aligning with the enduring efficacy of CBT-i (van der Zweerde et al., 2019), and the well-known post-treatment waning of relaxation benefits (Vambheim et al., 2021). Relaxation therapy is historically supported for insomnia treatment (Morin et al., 2006), but with low evidence quality (Edinger et al., 2021) and questioned by newer research (Furukawa et al., 2024). However, relaxation is effective in non-pharmacological pain management, (Vambheim et al., 2021), benefiting those with insomnia and daytime impairments (Lichstein et al., 2001), which could explain the positive impact on secondary measures in this study.

Insomnia-related beliefs and willingness exhibited significant time effects, indicating improvements from pretreatment to post-treatment and from post-treatment to follow-up, respectively, without notable differences between treatments. This raises the possibility that reductions in dysfunctional sleep beliefs, alongside increased willingness to accept sleep problems, may contribute to these outcomes, hinting at the possibility that diminishing the struggle against sleep difficulties is beneficial (Bothelius et al., 2015). These observations are consistent with recent reviews highlighting the association between decreased dysfunctional beliefs about insomnia and improved sleep outcomes (Nielson et al., 2023).

ICBT-i for chronic insomnia without comorbid pain shows high rates of non-completers, around 25 % (Zachariae et al., 2016). However, among patients with chronic pain, attrition is a problem not only in internet-based treatments; the non-completer rates in face-to-face chronic pain management programmes range from 10 % to over 50 % (Oosterhaven et al., 2019). Despite recommendations (Turk and Rudy, 1990), addressing non-completion in pain treatment studies remains overlooked (Oosterhaven et al., 2019).

The low level of treatment engagement could explain the lack of specific effects for ICBT-i, since it is assumed that sleep restriction and stimulus control would be the most effective techniques and only a minority of patients (41 %) finished module 2 where these were initiated, and even fewer likely used the techniques for one (31 %) or two (21 %) more weeks, which is likely needed to achieve a specific effect of ICBT-i.

These CBT-i techniques are challenging, potentially leading to lower adherence (Kyle et al., 2011). Conversely, relaxation therapy is considered the most acceptable behavioural intervention for insomnia (Edinger et al., 2021) due to immediate positive effects on stress and pain (Vambheim et al., 2021), offering negative reinforcement (Navratilova et al., 2012). Short sleep duration, higher depression rates (Ong et al., 2008), wake time after sleep onset, and expectations for sleep (Nam et al., 2023), have previously been linked to CBT-i dropout. In our study, a finding was that baseline ‘quality of life’ negatively correlated with non-initiation, suggesting it may be useful as a predictive marker.

The significant number of individuals declining participation or being lost to follow-up could be influenced by several factors. Firstly, individuals with chronic pain often experience high levels of fatigue and may find it challenging to commit to a structured treatment program like CBT-i. Additionally, there may be skepticism about the effectiveness of psychological interventions for pain-related insomnia, leading to reluctance to participate. While the online delivery of the treatment was intended to counteract barriers such as transportation and scheduling conflicts, other issues might still affect acceptability. For instance, some individuals may have limited access to or familiarity with digital technologies, which could hinder their participation. Furthermore, the commitment required for CBT-i is quite high, and might have been perceived as unacceptably burdensome. However, it is important to note that participants randomized to the control condition (applied relaxation) declined participation and were lost to follow-up to the same extent, suggesting that the issue might not be specific to CBT-i but rather related to the overall burden of participating in any additional treatment. Moreover, the acceptability of the intervention could be influenced by participants' beliefs and attitudes about sleep, particularly in the context of pain. Previous research has shown that inflexible beliefs about the interaction between pain and sleep can exacerbate insomnia and may reduce the effectiveness of interventions (Afolalu et al., 2016), and influence the attitudes towards treatments of insomnia (Ravyts et al., 2022). As pain-related beliefs and attitudes about sleep were not assessed in the current study we do not know if these mechanisms were at play, but this is an important aspect that should be further investigated in future studies.

Research indicates that integrating therapist telephone support, emphasizing sleep restriction/compression techniques in self-help CBT-i, can enhance adherence and outcome (Kaldo et al., 2015b). Lower engagement in the current study could stem from indirect support through asynchronous messaging, therapists' limited expertise in CBT-i and chronic pain management, and the inherent challenges in engaging this specific patient group as suggested in previous research.

### 4.1 Strengths and limitations

The current study's strengths include the use of a randomized controlled methodology, the real-world context of patient recruitment, and the employment of a rigorously evaluated treatment protocol. However, several limitations should be acknowledged. First, while participants were classified as having chronic benign pain according to IASP criteria, detailed diagnostic information was not collected from patient records, potentially limiting the generalizability of the findings to specific chronic pain conditions. Furthermore, although participants were required to maintain stable sleep and pain management, these conditions were not actively monitored, creating uncertainty about whether they were consistently upheld throughout the study. A potential weakness of the study is the challenge in detecting significant differences in AEs between the groups, which may be due to the small sample size and the high proportion of participants reporting no AEs.

A pivotal finding related to feasibility, marked by a high rate of non-initiation and overall limited treatment engagement, constrains the investigation into whether CBT-i can yield specific therapeutic benefits also for chronic pain patients. As there was high attrition in the groups (55–64 %) and a low between-group effect size on the primary outcome (ISI: d = 0.14), this is in stark contrast to the power estimation a priori; these facts are indications that the current study was likely underpowered to detect significant differences. It should also be underscored that the design of the study precludes conclusions regarding the putative mechanisms as mechanisms of change (Kazdin, 2007); this would, for example, require statistical analyses investigating temporality between changes in mechanisms and subsequent improvements in outcome measures. Nonetheless, a principal goal of this study was to enhance understanding of the challenges and feasibility within an actual clinical environment.

### 4.2 Future research

The findings underscore the necessity for enhanced understanding of customizing online interventions for those suffering from insomnia and chronic pain comorbidities. Future investigations should focus on strategies to increase treatment engagement, potentially incorporating qualitative research to delve deeper into perceptions of the pain-sleep nexus and quantitively exploring modifications in treatment content, delivery, and support in for example factorial design experiments. Blended interventions, which combine online and face-to-face components, have demonstrated efficacy in reducing dropout among various clinical groups (Erbe et al., 2017), and could be beneficial for this particular demographic as well.

The following are the supplementary data related to this article.

Supplementary Table S1 Content of the treatment manuals. Supplementary Table S1

Supplementary Table S2 Module completion for the participants in each treatment group. Supplementary Table S2

Supplementary Table S3 Completer analyses. Supplementary Table S3

## Funding sources

None.

## Declaration of competing interest

None.

## References

[bb0005] Afolalu E.F., Moore C., Ramlee F., Goodchild C.E., Tang N.K. (2016). Development of the Pain-Related Beliefs and Attitudes about Sleep (PBAS) Scale for the assessment and treatment of insomnia comorbid with chronic pain. J. Clin. Sleep Med..

[bb0010] Altena E., Ellis J., Camart N., Guichard K., Bastien C. (2023). Mechanisms of cognitive behavioural therapy for insomnia. J. Sleep Res..

[bb0015] Baglioni C., Altena E., Bjorvatn B., Blom K., Bothelius K., Devoto A., Espie C.A., Frase L., Gavriloff D., Tuuliki H., Hoflehner A., Hogl B., Holzinger B., Jarnefelt H., Jernelov S., Johann A.F., Lombardo C., Nissen C., Palagini L., Peeters G., Perlis M.L., Posner D., Schlarb A., Spiegelhalder K., Wichniak A., Riemann D. (2020). The European academy for cognitive behavioural therapy for insomnia: an initiative of the European Insomnia Network to promote implementation and dissemination of treatment. J. Sleep Res..

[bb0020] Barkham M., Bewick B., Mullin T., Gilbody S., Connell J., Cahill J., Mellor-Clark J., Richards D., Unsworth G., Evans C. (2013). The CORE-10: A short measure of psychological distress for routine use in the psychological therapies. Couns. Psychother. Res..

[bb0025] Bastien C.H., Vallieres A., Morin C.M. (2001). Validation of the insomnia severity index as an outcome measure for insomnia research. Sleep Med..

[bb0030] Berman A.H., Bergman H., Palmstierna T., Schlyter F. (2005). Evaluation of the drug use disorders identification test (DUDIT) in criminal justice and detoxification settings and in a Swedish population sample. Eur. Addict. Res..

[bb0035] Blom K., Jernelov S., Kraepelien M., Bergdahl M.O., Jungmarker K., Ankartjarn L., Lindefors N., Kaldo V. (2015). Internet treatment addressing either insomnia or depression, for patients with both diagnoses: a randomized trial. Sleep.

[bb0040] Blom K., Tarkian Tillgren H., Wiklund T., Danlycke E., Forssen M., Soderstrom A., Johansson R., Hesser H., Jernelov S., Lindefors N., Andersson G., Kaldo V. (2015). Internet-vs. group-delivered cognitive behavior therapy for insomnia: A randomized controlled non-inferiority trial. Behav. Res. Ther..

[bb0045] Blom K., Jernelov S., Lindefors N., Kaldo V. (2016). Facilitating and hindering factors in internet-delivered treatment for insomnia and depression. Internet Interv..

[bb0050] Blom K., Jernelov S., Ruck C., Lindefors N., Kaldo V. (2016). Three-year follow-up of insomnia and hypnotics after controlled internet treatment for insomnia. Sleep.

[bb0055] Blom K., Jernelov S., Ruck C., Lindefors N., Kaldo V. (2017). Three-year follow-up comparing cognitive behavioral therapy for depression to cognitive behavioral therapy for insomnia, for patients with both diagnoses. Sleep.

[bb0060] Blom K., Forsell E., Hellberg M., Svanborg C., Jernelov S., Kaldo V. (2024). Psychological treatment of comorbid insomnia and depression: A double-blind randomized placebo-controlled trial. Psychother. Psychosom..

[bb0065] Borkovec T.D., Nau S.D. (1972). Credibility of analogue therapy rationales. J. Behav. Ther. Exp. Psychiatry.

[bb0070] Bothelius K., Jernelov S., Fredrikson M., Mccracken L.M., Kaldo V. (2015). Measuring acceptance of sleep difficulties: the development of the sleep problem acceptance questionnaire. Sleep.

[bb0075] Breivik H., Collett B., Ventafridda V., Cohen R., Gallacher D. (2006). Survey of chronic pain in Europe: prevalence, impact on daily life, and treatment. Eur. J. Pain.

[bb0080] Cleeland C.S., Ryan K.M. (1994). Pain assessment: global use of the Brief Pain Inventory. Ann. Acad. Med. Singapore.

[bb0085] Climent-Sanz C., Valenzuela-Pascual F., Martinez-Navarro O., Blanco-Blanco J., Rubi-Carnacea F., Garcia-Martinez E., Soler-Gonzalez J., Barallat-Gimeno E., Gea-Sanchez M. (2022). Cognitive behavioral therapy for insomnia (CBT-i) in patients with fibromyalgia: a systematic review and meta-analysis. Disabil. Rehabil..

[bb0090] Cohen J. (1988).

[bb0095] Da Cunha Meneses F., Tingstam S. (2015).

[bb0100] Desalvo K.B., Muntner P. (2011). Discordance between physician and patient self-rated health and all-cause mortality. Ochsner J..

[bb0105] Desalvo K.B., Fisher W.P., Tran K., Bloser N., Merrill W., Peabody J. (2006). Assessing measurement properties of two single-item general health measures. Qual. Life Res..

[bb0110] Devilly G.J., Borkovec T.D. (2000). Psychometric properties of the credibility/expectancy questionnaire. J. Behav. Ther. Exp. Psychiatry.

[bb0115] Dworkin R.H., Turk D.C., Farrar J.T., Haythornthwaite J.A., Jensen M.P., Katz N.P., Kerns R.D., Stucki G., Allen R.R., Bellamy N., Carr D.B., Chandler J., Cowan P., Dionne R., Galer B.S., Hertz S., Jadad A.R., Kramer L.D., Manning D.C., Martin S., Mccormick C.G., Mcdermott M.P., Mcgrath P., Quessy S., Rappaport B.A., Robbins W., Robinson J.P., Rothman M., Royal M.A., Simon L., Stauffer J.W., Stein W., Tollett J., Wernicke J., Witter J., IMMPACT (2005). Core outcome measures for chronic pain clinical trials: IMMPACT recommendations. Pain.

[bb0120] Edinger J.D., Bonnet M.H., Bootzin R.R., Doghramji K., Dorsey C.M., Espie C.A., Jamieson A.O., Mccall W.V., Morin C.M., Stepanski E.J., American Academy of Sleep Medicine Work, G (2004). Derivation of research diagnostic criteria for insomnia: report of an American Academy of Sleep Medicine Work Group. Sleep.

[bb0125] Edinger J.D., Arnedt J.T., Bertisch S.M., Carney C.E., Harrington J.J., Lichstein K.L., Sateia M.J., Troxel W.M., Zhou E.S., Kazmi U., Heald J.L., Martin J.L. (2021). Behavioral and psychological treatments for chronic insomnia disorder in adults: an American Academy of Sleep Medicine systematic review, meta-analysis, and GRADE assessment. J. Clin. Sleep Med..

[bb0130] Erbe D., Eichert H.C., Riper H., Ebert D.D. (2017). Blending face-to-face and internet-based interventions for the treatment of mental disorders in adults: systematic review. J. Med. Internet Res..

[bb0135] Eriksson I., Unden A.L., Elofsson S. (2001). Self-rated health. Comparisons between three different measures. Results from a population study. Int. J. Epidemiol..

[bb0140] Espie C.A. (2002). Insomnia: conceptual issues in the development, persistence, and treatment of sleep disorder in adults. Annu. Rev. Psychol..

[bb0145] Espie C.A., Inglis S.J., Harvey L., Tessier S. (2000). Insomniacs’ attributions. Psychometric properties of the dysfunctional beliefs and attitudes about sleep scale and the sleep disturbance questionnaire. J. Psychosom. Res..

[bb0150] Forsell E., Jernelov S., Blom K., Kraepelien M., Svanborg C., Andersson G., Lindefors N., Kaldo V. (2019). Proof of concept for an adaptive treatment strategy to prevent failures in internet-delivered CBT: a single-blind randomized clinical trial with insomnia patients. Am. J. Psychiatry.

[bb0155] Furukawa Y., Sakata M., Yamamoto R., Nakajima S., Kikuchi S., Inoue M., Ito M., Noma H., Takashina H.N., Funada S., Ostinelli E.G., Furukawa T.A., Efthimiou O., Perlis M. (2024). Components and delivery formats of cognitive behavioral therapy for chronic insomnia in adults: a systematic review and component network meta-analysis. JAMA Psychiatry.

[bb0160] Geiger-Brown J.M., Rogers V.E., Liu W., Ludeman E.M., Downton K.D., Diaz-Abad M. (2015). Cognitive behavioral therapy in persons with comorbid insomnia: a meta-analysis. Sleep Med. Rev..

[bb0165] Harvey A.G. (2002). A cognitive model of insomnia. Behav. Res. Ther..

[bb0170] Hertenstein E., Trinca E., Wunderlin M., Schneider C.L., Zust M.A., Feher K.D., Su T., Straten A.V., Berger T., Baglioni C., Johann A., Spiegelhalder K., Riemann D., Feige B., Nissen C. (2022). Cognitive behavioral therapy for insomnia in patients with mental disorders and comorbid insomnia: A systematic review and meta-analysis. Sleep Med. Rev..

[bb0175] Husak A.J., Bair M.J. (2020). Chronic pain and sleep disturbances: a pragmatic review of their relationships, comorbidities, and treatments. Pain Med..

[bb0180] IASP (1986). Classification of chronic pain. Descriptions of chronic pain syndromes and definitions of pain terms. Prepared by the International Association for the Study of Pain, Subcommittee on Taxonomy. Pain Suppl.

[bb0185] Jansson-Frojmark M. (2014). The work and social adjustment scale as a measure of dysfunction in chronic insomnia: reliability and validity. Behav. Cogn. Psychother..

[bb0190] Jernelov S., Lekander M., Blom K., Rydh S., Ljotsson B., Axelsson J., Kaldo V. (2012). Efficacy of a behavioral self-help treatment with or without therapist guidance for co-morbid and primary insomnia–a randomized controlled trial. BMC Psychiatry.

[bb0195] Jernelov S., Blom K., Hentati Isacsson N., Bjurner P., Rosen A., Kraepelien M., Forsell E., Kaldo V. (2022). Very long-term outcome of cognitive behavioral therapy for insomnia: one- and ten-year follow-up of a randomized controlled trial. Cogn. Behav. Ther..

[bb0200] Johnson S.U., Ulvenes P.G., Oktedalen T., Hoffart A. (2019). Psychometric properties of the General Anxiety Disorder 7-Item (GAD-7) Scale in a heterogeneous psychiatric sample. Front. Psychol..

[bb0205] Kaldo V., Jernelov S., Blom K., Ljotsson B., Brodin M., Jorgensen M., Kraepelien M., Ruck C., Lindefors N. (2015). Guided internet cognitive behavioral therapy for insomnia compared to a control treatment - a randomized trial. Behav. Res. Ther..

[bb0210] Kaldo V., Ramnero J., Jernelov S. (2015). Involving clients in treatment methods: a neglected interaction in the therapeutic relationship. J. Consult. Clin. Psychol..

[bb0215] Kaldo V., Bothelius K., Blom K., Lindhe M., Larsson M., Karimi K., Melder S., Bondestam V., Ulfsparre C., Sternbrink K., Jernelov S. (2020). An open-ended primary-care group intervention for insomnia based on a self-help book - a randomized controlled trial and 4-year follow-up. J. Sleep Res..

[bb0220] Kazdin A.E. (2007). Mediators and mechanisms of change in psychotherapy research. Annu. Rev. Clin. Psychol..

[bb0225] Koffel E., Bramoweth A.D., Ulmer C.S. (2018). Increasing access to and utilization of cognitive behavioral therapy for insomnia (CBT-I): a narrative review. J. Gen. Intern. Med..

[bb0230] Kyle S.D., Morgan K., Spiegelhalder K., Espie C.A. (2011). No pain, no gain: an exploratory within-subjects mixed-methods evaluation of the patient experience of sleep restriction therapy (SRT) for insomnia. Sleep Med..

[bb0235] Lai L., Liu Y., Mccracken L.M., Li Y., Ren Z. (2023). The efficacy of acceptance and commitment therapy for chronic pain: a three-level meta-analysis and a trial sequential analysis of randomized controlled trials. Behav. Res. Ther..

[bb0240] Lichstein K.L., Riedel B.W., Wilson N.M., Lester K.W., Aguillard R.N. (2001). Relaxation and sleep compression for late-life insomnia: a placebo-controlled trial. J. Consult. Clin. Psychol..

[bb0245] Lindner P., Frykheden O., Forsstrom D., Andersson E., Ljotsson B., Hedman E., Andersson G., Carlbring P. (2016). The Brunnsviken Brief Quality of Life Scale (BBQ): development and psychometric evaluation. Cogn. Behav. Ther..

[bb0250] Lowe B., Decker O., Muller S., Brahler E., Schellberg D., Herzog W., Herzberg P.Y. (2008). Validation and standardization of the generalized anxiety disorder screener (GAD-7) in the general population. Med. Care.

[bb0255] Lundh L.G., Broman J.E. (2000). Insomnia as an interaction between sleep-interfering and sleep-interpreting processes. J. Psychosom. Res..

[bb0260] Mattila-Evenden M., Svanborg P., Gustavsson P., Asberg M. (1996). Determinants of self-rating and expert rating concordance in psychiatric out-patients, using the affective subscales of the CPRS. Acta Psychiatr. Scand..

[bb0265] McCracken L.M., Williams J.L., Tang N.K. (2011). Psychological flexibility may reduce insomnia in persons with chronic pain: a preliminary retrospective study. Pain Med..

[bb0270] McCrae C.S., Williams J., Roditi D., Anderson R., Mundt J.M., Miller M.B., Curtis A.F., Waxenberg L.B., Staud R., Berry R.B., Robinson M.E. (2019). Cognitive behavioral treatments for insomnia and pain in adults with comorbid chronic insomnia and fibromyalgia: clinical outcomes from the SPIN randomized controlled trial. Sleep.

[bb0275] Morin C.M. (1993).

[bb0280] Morin C.M., Bootzin R.R., Buysse D.J., Edinger J.D., Espie C.A., Lichstein K.L. (2006). Psychological and behavioral treatment of insomnia:update of the recent evidence (1998-2004). Sleep.

[bb0285] Mundt J.C., Marks I.M., Shear M.K., Greist J.H. (2002). The Work and Social Adjustment Scale: a simple measure of impairment in functioning. Br. J. Psychiatry.

[bb0290] Nam H., Chang J., Trockel M., Okajima I., Yang C.M., Chan N.Y., Li S., Suh S. (2023). Predictors of dropout in university students participating in an 8-week e-mail-based cognitive-behavioral therapy for insomnia intervention. Sleep Breath..

[bb0295] Navratilova E., Xie J.Y., Okun A., Qu C., Eyde N., Ci S., Ossipov M.H., King T., Fields H.L., Porreca F. (2012). Pain relief produces negative reinforcement through activation of mesolimbic reward-valuation circuitry. Proc. Natl. Acad. Sci. U. S. A..

[bb0300] Nielson S.A., Perez E., Soto P., Boyle J.T., Dzierzewski J.M. (2023). Challenging beliefs for quality sleep: a systematic review of maladaptive sleep beliefs and treatment outcomes following cognitive behavioral therapy for insomnia. Sleep Med. Rev..

[bb0310] Ong J.C., Kuo T.F., Manber R. (2008). Who is at risk for dropout from group cognitive-behavior therapy for insomnia?. J. Psychosom. Res..

[bb0315] Oosterhaven J., Wittink H., Mollema J., Kruitwagen C., Deville W. (2019). Predictors of dropout in interdisciplinary chronic pain management programmes: a systematic review. J. Rehabil. Med..

[bb0320] Pallesen S., Bjorvatn B., Nordhus I.H., Sivertsen B., Hjornevik M., Morin C.M. (2008). A new scale for measuring insomnia: the Bergen Insomnia Scale. Percept. Mot. Skills.

[bb0325] Parsons C.E., Zachariae R., Landberger C., Young K.S. (2021). How does cognitive behavioural therapy for insomnia work? A systematic review and meta-analysis of mediators of change. Clin. Psychol. Rev..

[bb0330] Qaseem A., Kansagara D., Forciea M.A., Cooke M., Denberg T.D., Clinical Guidelines Committee Of The American College of, P (2016). Management of chronic insomnia disorder in adults: A clinical practice guideline from the American College of Physicians. Ann. Intern. Med..

[bb0335] Ravyts S.G., Perez E., Dzierzewski J.M. (2022). Pain-related beliefs about sleep as a predictor of insomnia symptoms and treatment acceptability. Sleep Med..

[bb0340] Riemann D., Baglioni C., Bassetti C., Bjorvatn B., Dolenc Groselj L., Ellis J.G., Espie C.A., Garcia-Borreguero D., Gjerstad M., Goncalves M., Hertenstein E., Jansson-Frojmark M., Jennum P.J., Leger D., Nissen C., Parrino L., Paunio T., Pevernagie D., Verbraecken J., Weess H.G., Wichniak A., Zavalko I., Arnardottir E.S., Deleanu O.C., Strazisar B., Zoetmulder M., Spiegelhalder K. (2017). European guideline for the diagnosis and treatment of insomnia. J. Sleep Res..

[bb0345] Rutter L.A., Brown T.A. (2017). Psychometric properties of the Generalized Anxiety Disorder Scale-7 (GAD-7) in outpatients with anxiety and mood disorders. J. Psychopathol. Behav. Assess..

[bb0350] Saunders J.B., Aasland O.G., Babor T.F., De La Fuente J.R., Grant M. (1993). Development of the Alcohol Use Disorders Identification Test (AUDIT): WHO Collaborative Project on early detection of persons with harmful alcohol consumption—II. Addiction.

[bb0355] Schulz K.F., Grimes D.A. (2005). Multiplicity in randomised trials I: endpoints and treatments. Lancet.

[bb0360] Selvanathan J., Pham C., Nagappa M., Peng P.W.H., Englesakis M., Espie C.A., Morin C.M., Chung F. (2021). Cognitive behavioral therapy for insomnia in patients with chronic pain - a systematic review and meta-analysis of randomized controlled trials. Sleep Med. Rev..

[bb0365] Simon L., Steinmetz L., Feige B., Benz F., Spiegelhalder K., Baumeister H. (2023). Comparative efficacy of onsite, digital, and other settings for cognitive behavioral therapy for insomnia: a systematic review and network meta-analysis. Sci. Rep..

[bb0370] Smith M.T., Haythornthwaite J.A. (2004). How do sleep disturbance and chronic pain inter-relate? Insights from the longitudinal and cognitive-behavioral clinical trials literature. Sleep Med. Rev..

[bb0375] Spitzer R.L., Kroenke K., Williams J.B., Lowe B. (2006). A brief measure for assessing generalized anxiety disorder: the GAD-7. Arch. Intern. Med..

[bb0380] Streiner D.L. (2015). Best (but oft-forgotten) practices: the multiple problems of multiplicity-whether and how to correct for many statistical tests. Am. J. Clin. Nutr..

[bb0385] Sun Y., Laksono I., Selvanathan J., Saripella A., Nagappa M., Pham C., Englesakis M., Peng P., Morin C.M., Chung F. (2021). Prevalence of sleep disturbances in patients with chronic non-cancer pain: a systematic review and meta-analysis. Sleep Med. Rev..

[bb0390] Svanborg P., Asberg M. (1994). A new self-rating scale for depression and anxiety states based on the Comprehensive Psychopathological Rating Scale. Acta Psychiatr. Scand..

[bb0395] Svanborg P., Asberg M. (2001). A comparison between the Beck Depression Inventory (BDI) and the self-rating version of the Montgomery Asberg Depression Rating Scale (MADRS). J. Affect. Disord..

[bb0400] Turk D.C., Rudy T.E. (1990). Neglected factors in chronic pain treatment outcome studies–referral patterns, failure to enter treatment, and attrition. Pain.

[bb0405] Vambheim S.M., Kyllo T.M., Hegland S., Bystad M. (2021). Relaxation techniques as an intervention for chronic pain: a systematic review of randomized controlled trials. Heliyon.

[bb0410] Van Der Zweerde T., Bisdounis L., Kyle S.D., Lancee J., Van Straten A. (2019). Cognitive behavioral therapy for insomnia: A meta-analysis of long-term effects in controlled studies. Sleep Med. Rev..

[bb0415] Vos T., Allen C., Arora M., Barber R.M., Bhutta Z.A., Charlson F.J., Chen A.Z., Coggeshall M., Cornaby L., Dandona L., Dicker D.J., Dilegge T., Erskine H.E., Ferrari A.J., Fitzmaurice C., Fleming T., Forouzanfar M.H., Fullman N., Gething P.W., Goldberg E.M., Graetz N., Haagsma J.A., Hay S.I., Murray C.J.L. (2016). Global, regional, and national incidence, prevalence, and years lived with disability for 310 diseases and injuries, 1990–2015: a systematic analysis for the Global Burden of Disease Study 2015. Lancet.

[bb0420] Vugts M.A.P., Joosen M.C.W., Van Der Geer J.E., Zedlitz A., Vrijhoef H.J.M. (2018). The effectiveness of various computer-based interventions for patients with chronic pain or functional somatic syndromes: A systematic review and meta-analysis. PloS One.

[bb0425] Wiklund T., Molander P., Lindner P., Andersson G., Gerdle B., Dragioti E. (2022). Internet-delivered cognitive behavioral therapy for insomnia comorbid with chronic pain: randomized controlled trial. J. Med. Internet Res..

[bb0430] Wu J.Q., Appleman E.R., Salazar R.D., Ong J.C. (2015). Cognitive behavioral therapy for insomnia comorbid with psychiatric and medical conditions: A Meta-analysis. JAMA Intern. Med..

[bb0435] Zachariae R., Lyby M.S., Ritterband L.M., O’toole M.S. (2016). Efficacy of internet-delivered cognitive-behavioral therapy for insomnia - a systematic review and meta-analysis of randomized controlled trials. Sleep Med. Rev..

[bb0440] Zhou F.C., Yang Y., Wang Y.Y., Rao W.W., Zhang S.F., Zeng L.N., Zheng W., Ng C.H., Ungvari G.S., Zhang L., Xiang Y.T. (2020). Cognitive Behavioural therapy for insomnia monotherapy in patients with medical or psychiatric comorbidities: a meta-analysis of randomized controlled trials. Psychiatry Q..

