# Supplementary material for: Internet-based cognitive behavioural therapy for insomnia comorbid with chronic benign pain – A randomized controlled trial
Source: Internet Interv. 2024 Oct 14;38:100781. doi: 10.1016/j.invent.2024.100781 (PMC11533069; doi:10.1016/j.invent.2024.100781)
Supplement: Supplementary Table S1 — Content of the treatment manuals. [file mmc1.docx]

**Supplementary Table S1**

*Content of the treatment manuals.*

**ICBT-i (experimental group)**

| **Module** | **Content/Psychoeducation** | **Homework/Tasks** |
| --- | --- | --- |
| 1.a  Introduction | Facts about sleep and insomnia. Introduction to the sleep diary. | Sleep diary. |
| 1b.  CBT model | Information about cognitive behavioral therapy (CBT), CBT model for insomnia and facts about sleep medication. | Sleep diary.  Goal formulation.  Make a plan for sleep medication (if taking any). |
| 1c.  Tired or Sleepy - Myths | Understanding the difference between being tired and sleepy. Learning about sleep myths. | Sleep diary.  Worksheet: My fatigue.  Calculate sleep duration and sleep efficiency. |
| 2a.  Sleep Rhythm | Sleep compression. Relaxation and visualization exercises. | Sleep diary.  Calculate sleep compression window and begin sleep compression.  Perform quick relaxation and visualization exercises. |
| 2b.  The bed’s signals | Information about stimulus control and strategies to use when feeling tired. | Sleep diary.  Sleep compression.  Stimulus control.  Exercise: Turn away the clock. |
| 3.  Daytime and Bedtime | Facts about daylight and daytime activities. | Sleep diary  Sleep compression.  Stimulus control.  Worksheet: Assess and plan your daytime and bedtime routines. |
| 4.  Here is where you are | Mindfulness and Acceptance. | Sleep diary  Sleep compression.  Stimulus control.  Continue to map and plan your daytime and bedtime routines. Exercise: Mindfulness and acceptance. |
| 5.  Managing Thoughts | Identify myths and thought traps. Challenge automatic thoughts. Behavioral experiments. | Sleep diary.  Sleep compression.  Stimulus control.  Continue to map and plan your daytime and bedtime routines. Exercise: Mindfulness and acceptance.  Exercise: Find and manage your thoughts. |
| 6.  Sleep Hygiene - Keeping Sleep Clean | Sleep hygiene. Naps. | Sleep diary.  Sleep compression.  Stimulus control.  Continue to map and plan your daytime and bedtime routines.  Exercise: Mindfulness and acceptance.  Exercise: Find and manage your thoughts. Reflect on internal and external sleep hygiene. |
| 7.  More Thoughts, More Acceptance | Module for deepening and working further with previous tools (relaxation exercises, mindfulness, or thoughts). | Sleep diary.  Sleep compression.  Stimulus control.  Continue to map and plan your daytime and bedtime routines.  Exercise: Mindfulness and acceptance.  Exercise: Managing thoughts.  Worksheet: Become your own sleep detective. Choose an advanced exercise. |
| 8.  So Far and Ahead | Plan ahead. Evaluate. | Sleep diary.  Sleep compression.  Stimulus control.  Work on daytime and bedtime routines.  Exercise: Mindfulness and acceptance.  Exercise: Managing thoughts.  Worksheet: Become your own sleep detective.  Worksheet: My plan ahead. |

**IAR (Control Group)**

| **Module** | **Content/Psychoeducation** | **Homework/Tasks** |
| --- | --- | --- |
| 1.  Introduction | About internet treatment and the treatment structure. |  |
| 2.  Extended Relaxation | Receive information about how extended relaxation works. | Practice extended relaxation twice a day for 5 days and record them. |
| 3.  Extended Relaxation in the Whole Body | Receive information about abdominal breathing and how full-body extended relaxation works. | Practice abdominal breathing. Practice full-body extended relaxation twice a day for 5 days and record them. |
| 4.  Short Relaxation | Receive information about short relaxation. | Practice short relaxation twice a day for the week and record the exercises. |
| 5.  Conditioned Relaxation | Learn what conditioned relaxation is and use a relaxation word during relaxation. | Practice conditioned relaxation twice a day for the week and record the exercises. |
| 6.  Active Relaxation | Learn what active relaxation is and get instructions on how active relaxation works. | Practice active relaxation twice a day for the week and record the exercises. |
| 7a.  Quick Relaxation | Practice relaxing in natural situations and reduce the time it takes to relax. | Practice quick relaxation twice a day for 3 days and record the relaxation exercises. |
| 7b.  Quick Relaxation in More Challenging Situations | Practice quick relaxation in more challenging situations. | Practice quick relaxation 15-20 times per day and record them. |
| 8.  Recap and Maintenance | Summary. Information about setbacks and relapses. Information about maintenance. | Create a maintenance plan. |

*Note*. ICBT-i = Internet-based Cognitive Behavioural Therapy for Insomnia, IAR = Internet-based Applied Relaxation.
