# Supplementary material for: Internet-based cognitive behavioural therapy for insomnia comorbid with chronic benign pain – A randomized controlled trial
Source: Internet Interv. 2024 Oct 14;38:100781. doi: 10.1016/j.invent.2024.100781 (PMC11533069; doi:10.1016/j.invent.2024.100781)
Supplement: Supplementary Table S2 — Module completion for the participants in each treatment group. [file mmc2.docx]

**Supplementary Table S2**

*Module completion for the participants in each treatment group.*

|  | ICBT-i (n=42) | IAR (n=43) |
| --- | --- | --- |
|  | n (%) | n (%) |
| 0 modules | 13 (31.0) | 14 (32.6) |
| 1 module | 11 (26.2) | 12 (27.9) |
| 2 modules | 4 (9.5) | 4 (9.3) |
| 3 modules | 4 (9.5) | 1 (2.3) |
| 4 modules | 2 (4.8) | 0 (0) |
| 5 modules | 2 (4.8) | 2 (4.7) |
| 6 modules | 1 (2.4) | 2 (4.7) |
| 7 modules | 3 (7.1) | 4 (9.3) |
| 8 modules | 2 (4.8) | 4 (9.3) |

*Note*. ICBT-i = Internet-based Cognitive Behavioural Therapy for Insomnia, IAR = Internet-based Applied Relaxation.
