# Supplementary material for: Internet-based cognitive behavioural therapy for insomnia comorbid with chronic benign pain – A randomized controlled trial
Source: Internet Interv. 2024 Oct 14;38:100781. doi: 10.1016/j.invent.2024.100781 (PMC11533069; doi:10.1016/j.invent.2024.100781)
Supplement: Supplementary Table S3 — Completer analyses. [file mmc3.docx]

**Supplementary Table S3**

*Completer analyses*

Ten individuals in the ICBT-i group and 12 in the IAR group completed four modules or more. These 22 participants were classified as treatment completers, since they had completed at least half of the modules and the ICBT-i group had been administered the two most vital treatment components, i.e., sleep restriction and stimulus control. To estimate the efficacy of ICBT-i and IAR among completers on the primary outcome, insomnia severity (the ISI), see table below for estimated marginal means and standard errors, between-group effect sizes were estimated at post-treatment and follow-up for completers. Between-group effect sizes were similar for ICBT-I and IAR at post-treatment among both completers and the total sample. For completers, Cohen’s *d* was 0.00 (95% CI -0.92–0.92) and for the total sample, *d* was 0.14 (95% CI -0.57–0.29). However, ICBT-i resulted in larger effect sizes at follow-up relative to IAR for completers (*d* = 0.74, 95% CI -0.13–1.79) compared with the total sample (*d* = 0.22, 95% CI -0.21–0.66). Taken as a whole, the completers versus total sample comparisons suggest a pattern in which ICBT-i completers displayed a more favourable outcome than IAR completers at follow-up but not at post-treatment.

|  | Group | Pre-treatment | Post-treatment | Follow-up |
| --- | --- | --- | --- | --- |
|  |  | M (SD) | M (SD) | M (SD) |
| ISI | ICBT-i | 20.4 (3.0) | 13.4 (7.8) | 10.7 (3.9) |
|  | IAR | 19.3 (4.2) | 13.4 (4.7) | 15.2 (6.9) |

*Note*. ICBT-i = Internet-based Cognitive Behavioural Therapy for Insomnia, IAR = Internet-based Applied Relaxation.
